# The allocation of resources in the care for patients with panic disorder in Germany: an excess cost analysis informing policy and science

**DOI:** 10.1186/s12962-019-0177-4

**Published:** 2019-04-27

**Authors:** Christian Brettschneider, Florian Bleibler, Thomas S. Hiller, Alexander Konnopka, Jörg Breitbart, Jürgen Margraf, Jochen Gensichen, Hans-Helmut König, for the Jena-PARADISE Study-Group, Wolfgang Blank, Florian Bleibler, Jörg Breitbart, Christian Brettschneider, Antje Freytag, Jochen Gensichen, Thomas S. Hiller, Heike Hoyer, Bert Huenges, Hans-Helmut König, Armin Mainz, Jürgen Margraf, Sylvia Sänger, Mercedes Schelle, Konrad Schmidt, Nico Schneider, Elisabeth Schöne, Ulrike Schumacher, Michael Sommer, Tobias Teismann, Paul Thiel, Michel Wensing

**Affiliations:** 1 Department of Health Economics and Health Services Research, Hamburg Center for Health Economics, University Medical Center Hamburg-Eppendorf, Martinistraße 52, 20246 Hamburg, Germany; 2 Institute of General Practice & Family Medicine, Jena University Hospital, Friedrich-Schiller-University, Bachstrasse 18, 07743 Jena, Germany; 3 Mental Health Research and Treatment Center, Ruhr-University Bochum, Massenbergstrasse 9-13, 44787 Bochum, Germany; 4 Institute of General Practice and Family Medicine, University Hospital of Ludwig-Maximilians-University Munich, Pettenkoferstraße 10, 80336 Munich, Germany

**Keywords:** Panic disorder, Agoraphobia, Costs, Economic burden, Absenteeism

## Abstract

**Background:**

Panic disorder is a mental disorder of high prevalence, which frequently co-occurs with agoraphobia. There is a lack of studies measuring excess costs of panic disorder patients with and without agoraphobia. We compared costs of panic disorder patients with or without agoraphobia with costs of the anxiety-free population in Germany.

**Methods:**

Primary data from a cluster-randomized trial of adults suffering from panic disorder (n = 419) and from a representative survey of the German general population (N = 5005) were collected between 2012 and 2014. Missing data from the cluster-randomized trial were first imputed by multiple imputation using chained equations and subsequently balanced with the data from the survey by Entropy Balancing. The societal perspective was chosen. Excess costs were calculated by generalized linear models and two-part-models.

**Results:**

Entropy Balancing led to an exact match between the groups. We found 6-month total excess costs of 3220€ (95% CI 1917€–4522€) for panic disorder patients without agoraphobia and of 3943€ (95% CI 2950€–4936€) for patient with agoraphobia. Panic disorder patients with or without agoraphobia had significantly higher costs for psychotherapy, general practitioners, general hospital stays and informal care Indirect costs accounted for approximately 60% of the total excess costs.

**Conclusions:**

Panic disorder with or without agoraphobia is associated with significant excess costs. Agoraphobia changes the pattern of resource utilization. Especially indirect costs are relevant. Agoraphobia influences resource utilization in the inpatient sector.

*Trial registration* ISRCTN64669297

## Introduction

Panic disorder, defined as recurrent, unpredictable panic attacks associated with feelings of unreality, chest pain, palpitations, choking sensations, and dizziness [1], poses a major challenge to the health care system. According to the representative DEGS1-MH study, in Germany the 12-month prevalence of panic disorder (with or without agoraphobia) is approximately 2.0% in the general adult population, with women being affected twice as often as men (prevalence rate of 2.8% vs. 1.2%) [2]. This study considered the German adult population aged 18 to 79. Based on data of the German Statistical Office, this age group consisted of around 64 million people in 2016 [3]. This means that around 1.28 million people in Germany suffered from panic disorder (with or without agoraphobia). Comorbidity among anxiety disorders is very common, with the highest rates found in panic disorder [4]. Agoraphobia is a frequent comorbidity in panic disorder patients. There is evidence that approximately 35% to 65% of the panic disorder patients are affected by agoraphobia [5].

The evidence highlights the challenge posed by frequency. Further evidence underlines the challenge posed by health burden. A study by Saarni et al. [6] showed that health-related quality of life was decreased for panic disorder patients as well as for patients with agoraphobia compared to individuals without a mental disorder [based on a composite international diagnostic interview (CIDI)]. Additionally, Beard et al. [7] reported that panic disorder patients with agoraphobia had lower physical and mental component scores in the Short-Form 36 compared to panic disorder patients without agoraphobia. Both studies indicate that the health burden of panic disorder with and without agoraphobia is high.

In addition, several studies emphasized the challenges to health care delivery and financing. Prospective longitudinal studies have shown that misinterpretation of bodily sensations and health anxiety are important factors which might contribute to elevated help-seeking in the health care system [8, 9]. Furthermore, the economic burden of panic disorder has been addressed by several international studies [10]. However, only one study from the Netherlands analysed the economic burden of panic disorder with a special view on agoraphobia [11]. The authors of this study differentiated between full-blown and subthreshold panic disorder with or without agoraphobia. They found that in case of a panic disorder agoraphobia raises the annual costs from around €12,000 to €15,000, while in case of subthreshold panic disorder the increase is even more pronounced, from around €6700 to nearly €17,000. Based on these results, we expect that in our study patients with a comorbid agoraphobia will have higher costs, too. However, due to methodological differences, we abstain from deriving assumptions about the amount of the difference and will not be able to compare the findings of Batelaan et al. to our results.

The special need to address these challenges has been recognized by German researchers and disease-specific interventions have been developed, implemented and assessed, e.g. the Jena-Paradise study (ISRCTN64669297). However, there is no scientific evidence regarding the impact of panic disorder (with or without agoraphobia) on health care delivery and costs in Germany. This information is pivotal to answer different questions, like “In which sectors are resource mainly utilized?”, “Which sectors need to be addressed by political and medical interventions?” and “Has an intervention been successful in addressing these sectors?”.

In this study, we will present this pivotal information by performing an excess cost analysis of patients with panic disorder (with or without agoraphobia) in comparison to a representative sample of the German adult population. By doing this, we will inform decision-makers on the allocation of health care resources, give them information to govern this allocation, support researchers in the development of future interventions and enable the interpretation of the impact of these interventions on the delivery of health care services.

## Methods

### Study population

Two samples are needed to estimate excess costs. The first sample consists of patients who suffer from the disorder of interest. The second sample is a population not affected by the disorder. In any other characteristic, the samples must be comparable. For this reason, we balanced the data of patients diagnosed with PD or PDA and data of individuals without anxiety disorders.

Patient data were collected in a cluster-randomized controlled trial including patients with panic disorder (PD) or panic disorder and agoraphobia (PDA) [12] (Current Controlled Trials: ISCRTN64669297). Patients had to be at least 18 years, diagnosed with PD (ICD-10; F41.0) or PDA (ICD-10; F40.01) by a GP-led clinical interview, scored at least 8 points on the Overall Anxiety Severity and Impairment Scale (OASIS) [13], and had a minimum of two positive answers on the panic module of the ‘Patient Health Questionnaire’ (PHQ) [14, 15]. Patients were excluded if they had one of the following conditions: suicidal tendencies, psychotic or substance-related disorders, severe physical illness, pregnancy, or if they were currently under psychotherapeutic treatment. In order to estimate excess costs, we used data from the baseline assessment, which included information about resource utilisation in the last 6 months before the assessment, sociodemographic characteristics and information on comorbidity. 419 patients were included in the study, 315 (75%) were diagnosed with PDA and 104 (25%) with PD. To handle missing values (44.6% of all patients had at least one missing value), we applied Multiple Imputation by Chained Equations [16]. In total 40 datasets (m = 40) [17] were generated by predicted mean matching [18] using sociodemographic characteristics, comorbidities, disease-specific measures, and health care utilisation as covariates in the imputation models.

The dataset used for comparison came from a telephone survey [19]. The sample is representative of the total German-speaking population aged 18 years and older. The interview included questions regarding health care utilisation in the last 6 months, the disease history (see Table 2), the PHQ-4 [20, 21] as a screening tool for anxiety disorders or depression, the ISR-S [21, 22] as a screening tool for somatoform disorders as well as sociodemographic questions. In total, 5005 individuals completed the telephone interview. A description of the dataset can be found elsewhere [19]. As individuals in this dataset should serve as an anxiety free control group in our analysis, we excluded individuals with anxiety disorders (n = 265) or with a score of at least 3 on the PHQ-4 anxiety module [21] (n = 1448). Furthermore, 78 individuals with missing values were removed from the dataset, as it was not possible to impute both datasets. Overall, 3214 individuals remained in the dataset as “anxiety free group (AF)” for further analysis.

### Health care utilisation and costs

Both studies were conducted by researchers from the same academic department by using approaches to data collection standardized in the department. The description of the single services as well as the units of measurement (contacts, days, hours) were comparable in both studies. In both datasets, information on health care resource use in three different health care sectors was collected. In the *outpatient sector*, visits to a psychiatrist/neurologist, psychologist/psychotherapist, general practitioner and other medical specialists were considered. In the *inpatient sector*, days in general, psychiatric and rehabilitation hospitals were assessed. In the home care sector, received hours of professional and informal caregiving (unpaid support and care provided by family members and friends) were measured. Additionally, disease-related days away from work (absenteeism) were recorded.

We applied the societal perspective [23]. Direct health care costs, i.e. costs directly related to treatments of diseases, were determined by valuing resource use data with German unit costs [24]. Indirect costs, i.e. productivity losses due to disease-related absence from work, were determined applying the human capital approach [25], valuing sick leave days with an average daily wage rate (including full- and part-time work, corrected for employees’ share of social contributions) [26]. All applied unit costs are reported in Table 1. Informal care has been monetarily valued by the replacement cost approach. The reference year of the cost calculation was 2012. If unit costs were not available for the base year, values were inflated to the year 2012 using the German consumer price index [27]. To avoid influence of outliners with high costs on the results we excluded individuals who had total healthcare costs above the 99th percentile (33 individuals in the AF group, 8 individuals in the PDA and 2 individuals in the PD group).

**Table 1:** Unit costs for different resource uses in 2012 Euros

Cost category	Unit	Price per unit (€)
Direct costs		
Outpatient sector		
Psychiatrist/neurologist	Per visit	45.60
Psychologist/psychotherapist	Per visit	79.61
General practitioner	Per visit	20.45
Other medical specialists	Per visit	35.37
Inpatient sector		
General hospital	Per day	587.18
Psychiatric hospital	Per day	346.36
Rehabilitation hospital	Per day	124.24
Home care sector		
Professional care	Per hour	29.36
Informal care	Per hour	18.33
Indirect costs		
Absenteeism	Per day	245.6

Sources: direct costs: [24, 27]; indirect costs: 30.7€/h [26] assuming 8 h/day

### Statistical analysis

In order to estimate excess costs of patients with PD and PDA in comparison to AF individuals, we applied a two-stage approach, comprising the *pre*-*processing* and the *estimation stage*. The main analyses of these study were performed based on the imputed dataset. However, to assess the influence of the imputation method (MICE) on the results we re-ran sensitivity analyses based on the original dataset, which is the dataset without imputation. Additionally, we performed sensitivity analyses based on a sample including the cost outliers to assess the influence of the approach in our main analysis. All analyses were conducted in Stata 14 (StataCorp, Texas, USA).

The *pre*-*processing stage* was necessary because the three groups differed in terms of their covariates and were therefore not directly comparable. Hence, we reweighted the AF group on the PD and PDA group separately, applying the reweighting method of Entropy Balancing [28]. In Entropy Balancing covariates of a target group will be made comparable to those of a comparison group in reference to predefined moments. In case of our study, all available covariates (Table 2) were included and the predefined moments were the mean and the variance of the target group. The process is successful if the value of the adjusted moments of the covariates in the target group are similar to the corresponding moments of the comparison group, which means that the adjusted value of all included covariates is in a predefined area of tolerable deviation. In case of our study this tolerance level was set to 0.005, a rigorous level of tolerance (the default level set in STATA is 0.015). Entropy balancing works in such a way that every observation in the target group receives a balancing weight. This balancing weight represents the number of times the specific observation is considered in the process of adjusting the included covariates in terms of the predefined moments. The algorithm varies the balancing weights until the moments of all included covariates of the target group are comparable to the moments of the comparison group [28]. A reweighted dataset is similar to a randomized trial, where both groups are expected to have no systematic difference in observed baseline characteristics. We chose this approach as it does not exclude members of the target group, reaches a higher degree of balance in the covariates than other approaches, and does not require manual adjustments by the scientist, which is a source of computational delay and human error. Entropy Balancing was performed for each of the imputed dataset (m = 40) separately. To perform the method we used a user written Stata program (Ebalance) by Hainmueller et al. [29].

**Table 2:** Pre- and post-balanced covariates

Covariates	Pre-balancing			Post-balancing			
	AF	PD	PDA	AF^a^	PD	AF^b^	PDA
	(n = 3181)	(n = 102)	(n = 307)	(n = 3181)	(n = 102)	(n = 3181)	(n = 307)
Age (mean)	56.239	46.069	46.055	45.941	46.069	46.015	46.055
Gender (female %)	0.489	0.686	0.765	0.684	0.686	0.765	0.765
Person in partnership (%)	0.455	0.520	0.495	0.518	0.520	0.495	0.495
Average persons in household	2.012	2.451	2.454	2.444	2.451	2.451	2.454
Educational level (%)							
None	0.007	0.039	0.036	0.050	0.039	0.036	0.036
Low	0.273	0.245	0.287	0.245	0.245	0.287	0.287
Median	0.307	0.343	0.420	0.343	0.343	0.420	0.420
High	0.413	0.363	0.257	0.362	0.363	0.257	0.257
Employment rate (%)	0.458	0.588	0.616	0.586	0.588	0.615	0.616
Physical comorbidities (%)							
Lung disease	0.141	0.167	0.137	0.167	0.167	0.137	0.137
Joint disease	0.295	0.069	0.143	0.069	0.069	0.143	0.143
Metabolic disease	0.239	0.265	0.306	0.265	0.265	0.306	0.306
Diabetes	0.107	0.029	0.101	0.029	0.029	0.101	0.101
Chronic pain	0.238	0.039	0.046	0.039	0.039	0.046	0.046
Gastrointestinal dis.	0.162	0.206	0.238	0.206	0.206	0.238	0.238
Cancer	0.094	0.020	0.033	0.020	0.020	0.033	0.033
Cardiovascular dis.	0.328	0.275	0.410	0.274	0.275	0.410	0.410
Skin disease	0.124	0.049	0.078	0.049	0.049	0.078	0.078
Osteoporosis	0.066	0.049	0.016	0.049	0.049	0.016	0.016
Mental comorbidities (%)							
Posttraumatic stress disorder	0.019	0.010	0.013	0.010	0.010	0.013	0.013
Somatoform disorder	0.032	0.088	0.094	0.088	0.088	0.094	0.094
Eating disorder	0.014	0.000	0.003	0.000	0.000	0.003	0.003
ADHS	0.004	0.000	0.000	0.000	0.000	0.000	0.000
Substance abuse	0.016	0.010	0.029	0.010	0.010	0.029	0.029
Psychoses	0.005	0.000	0.003	0.000	0.000	0.003	0.003
Depression	0.039	0.137	0.251	0.137	0.137	0.251	0.251

*AF* anxiety free, *PD* panic disorder, *PDA* panic disorder with agoraphobia

^a^AF were balanced on covariate structure of the PD sample

^b^AF were balanced on covariate structure of the PDA sample

In the *estimation stage*, we estimated mean cost differences (excess costs) between the groups for each cost category (Table 3). As costs cannot reach negative values, are often skewed, and sometimes have a large number of ‘zero’ values [30], the choice of an appropriate statistical model is an important step in modelling costs. For cost categories with a small number of ‘zeros’, costs were modelled using a generalized linear model (GLM) with gamma family and log-link function [31]. For cost categories with a large number of ‘zeros’, a two-part (TP) model approach was applied [32]. In the first part of the TP-model we used a logit model in order to estimate the probability of positive costs, in the second part we used a GLM with gamma family and log-link function to estimate costs in patients with positive costs [32]. The TP-model was estimated using the user-written Stata command twopm by Belotti et al. [32]. Mean differences in costs were obtained by calculating margins [32], which means we predicted the cost difference between the groups based on the results of the previously run model under consideration of the specific cost distribution. These results are referred to as excess costs in the further course of this article. As the twopm and margin command is not supported under Stata’s “mi estimate option”, we estimated 40 separate regression models (TPMs and GLMs) and combined the results using Rubin’s pooling rules for multiple imputed datasets [33]. All regressions were conducted as weighted regressions using the balancing weights from the pre-processing stage [28]. The only independent variable in all regressions was the group variable [PD or PDA (yes/no)]. We did not include further covariates into regression analyses as all observed covariates were already included in the Entropy balancing and would not have any further effect on averages of cost estimates [34].

**Table 3:** Average per capita 6-month costs of AF, PD and PDA for different cost categories in 2012 Euros

	AF^a^	PD	AF^b^	PDA
	(n = 3181)	(n = 102)	(n = 3181)	(n = 307)
	Mean (SD)	Mean (SD)	Mean (SD)	Mean (SD)
Direct costs				
Outpatient sector				
Psychiatrist/neurologist	5 (2)	24 (14)	8 (3)	23 (5)
Psychologist/psychotherapist	17 (4)	106 (35)	21 (5)	139 (20)
General practitioner	34 (1)	164 (13)	40 (2)	169 (9)
Other medical specialists	120 (8)	140 (19)	138 (12)	120 (11)
Inpatient sector				
General hospital	251 (46)	771 (197)	282 (86)	556 (106)
Psychiatric hospital	4 (2)	28 (66)	7 (4)	338 (141)
Rehabilitation hospital	28 (12)	137 (135)	35 (15)	221 (91)
Home care sector				
Professional care	12 (4)	1 (2)	7 (3)	2 (2)
Informal care	71 (25)	438 (170)	95 (31)	673 (149)
Total direct cost	542 (61)	1809 (354)	631 (103)	2242 (298)
Total indirect cost (Absenteeism)	427 (73)	2380 (531)	498 (99)	2830 (355)
Total cost	969 (110)	4189 (658)	1129 (184)	5072 (473)

AF costs are post balanced estimates

*AF* anxiety free, *PD* panic disorder, *PDA* panic disorder with agoraphobia, *SD* standard derivation

^a^AF were balanced on covariate structure of the PD sample

^b^AF were balanced on covariate structure of the PDA sample

## Results

The pre- and post-balancing results of the demographic and clinical characteristics for individuals from the AF population and patients with PDor PDA are shown in Table 2. By using entropy balancing we reached a high degree of concordance between the groups, in all included covariates.

The results of the cost-analysis of the AF group in comparison to the PD group are shown in Tables 3 and 4. As a main result, we found average (SD) 6-month total costs of 969€ (110€) in the AF group and 4189€ (658€) in the PD group, resulting in significant 6-month total excess costs of 3220€ (95% CI 1917€–4522€) for patients with PD in comparison to AF individuals. About 1953€ (95% CI 909–2996€) of the 6-month total excess costs were indirect costs due to absenteeism. This corresponds to 61% of the total excess costs. The largest share of the direct excess costs of 1267€ (95% CI 565€–1969€) was incurred by statistically significant general hospital excess costs of 520€ (95% CI 124€–916€) and excess costs due to informal care of 367€ (95% CI 31€–703€). In the outpatient sector we found significant excess costs of 130€ (95% CI 104€–155€) caused by visits to general practitioner and excess costs of 89€ (95% CI 19€–159€) for psychologist or psychotherapist visits. We found no statistically significant excess costs for treatments by other medical specialists, psychiatrists/neurologists, and for treatments in psychiatric or rehabilitation hospitals.

**Table 4:** 6-month per capita excess cost of PD and PDA for different cost categories in 2012 Euros

	AF^a^ group vs. PD group			AF^b^ group vs. PDA group		
	Excess	95% CI	p-value	Excess	95% CI	p-value
Direct costs						
Outpatient sector						
Psychiatrist/neurologist	19	(− 9 to 47)	0.191	15	(2 to 27)	0.020
Psychologist/psychotherapist	89	(19 to 159)	0.012	119	(78 to 159)	< 0.000
General practitioner	130	(104 to 155)	< 0.000	130	(112 to 148)	< 0.000
Other medical specialists	21	(− 20 to 61)	0.313	− 18	(− 51 to 14)	0.261
Inpatient sector						
General hospital	520	(124 to 916)	0.010	275	(14 to 535)	0.039
Psychiatric hospital	24	(− 107 to 155)	0.718	331	(53 to 610)	0.020
Rehabilitation hospital	109	(− 156 to 374)	0.422	187	(6 to 368)	0.043
Home care sector						
Professional care	− 11	n. a	n. a	− 5	n. a	n. a
Informal care	367	(31 to 703)	0.032	579	(281 to 876)	< 0.000
Total direct cost^a^	1267	(565 to 1969)	< 0.000	1612	(994 to 2229)	< 0.000
Total indirect cost (Absenteeism)	1953	(909 to 2996)	< 0.000	2331	(1617 to 3045)	< 0.000
Total cost	3220	(1917 to 4522)	< 0.000	3943	(2950 to 4936)	< 0.000

AF costs are post balanced estimates

*AF* anxiety free, *PD* panic disorder, *PDA* panic disorder with agoraphobia

^a^AF were balanced on covariate structure of the PD sample

^b^AF were balanced on covariate structure of the PDA sample

As shown in Tables 3 and 4, patients with PDA had average (SD) 6-month total costs of 5072€ (473€) compared to 1129€ (184€) in AF individuals, resulting in statistically significant total excess costs of 3943€ (95% CI 2950€–4936€). The share of indirect cost (2331€ (95% CI 1617€–3045€)) was 59%. The largest share of the direct excess costs (1612€ (95% CI 994€–2229€)) in the PDA group were excess costs of 579€ (95% CI 281€–876€) for informal care. In the inpatient sector, statistically significant excess costs incurred for psychiatric (331€ (95% CI 53€–610€)), general (275€ (95% CI 14€–535€)) and rehabilitation hospital stays (187€ (95% CI 6€–368€)). In the outpatient sector, excess costs incurred for treatments by general practitioners (130€ (95% CI 112€–148€) and psychologists or psychotherapists (119€ (95% CI 78€–159€)). Low but statistically significant excess costs were found for treatments by psychologists or psychotherapists with 15€ (95% CI 2€–27€). Excess costs for treatment by other medical specialists were not statistically significant.

The sensitivity analyses on the imputation of missing values led to results comparable to the results of the main analyses (Appendix Table 5). The amount of the excess costs decreased in several cost categories and the CI of the costs for inpatient rehabilitation services crossed the 0€ threshold, leading to an insignificant group difference. The sensitivity analyses considering the cost outliers showed no noticeable deviating results in the comparison of AF and PD. However, in the comparison of AF and PDA, we observed that in the inpatient sector the cost differences in general and psychiatric hospital services were not statistically significant anymore. The small sample size of the PDA group and the high cost in the inpatient sector could be an explanation for this.

## Discussion

Our analysis showed that PD and PDA raised health care costs (direct costs) and productivity losses (indirect costs) substantially. Around 60% of total costs were caused by productivity losses due to absenteeism. In the first instance, productivity losses are a loss to the economy and society and not necessarily influenced by resource allocation. However, scrutinizing the composition of excess cost for health care resources, our results indicate that there could be an interconnection between resource allocation and absenteeism. The utilization of general hospital services by PD patients is the resource category associated with the largest difference in costs. For PDA patients only the difference in informal care was larger. It is obvious that a higher utilization of hospital services goes along with a higher degree of absenteeism. This is an important aim for policy and medical interventions. Measures structured to shift the emphasis of treatment from the inpatient to the outpatient sector could not only result in lower health care costs but also in smaller losses of economic productivity. However, in this context it is indicated to differentiate between PD and PDA. While PDA showed an increase of inpatient service utilization in all subtypes of hospitals—and the largest increase for utilization of psychiatric hospitals—, PD showed a pronounced increase for general hospital services exclusively.

This pronounced excess utilization of general hospital services by PD patients can be interpreted as a direct result of a panic attack, which manifests with distinct somatic symptoms like chest pain, dizziness and breathing difficulties [1]. These symptoms can lead to the assumption of a severe physical and maybe even life-threatening disease. In this situation, people will be admitted to a hospital, maybe as emergency, and will first underwent a thorough evaluation in the inpatient setting. This process utilized resources in an unproductive way. There are different explanations for this. For example, (a) the patient was not diagnosed with a panic disorder, (b) the patient is not informed about the nature of his disorder, (c) the clinical staff is not qualified enough to differentiate between a heart attack and a panic attack with comparable symptoms or (d) clinical pathways demand that a patient showing these symptoms has to be hospitalized for a rigorous examination. Although some of these problems have been addressed before [35], the reasons for the hospitalization of PD patients in a general hospital has to be evaluated more thoroughly in further research.

In case of PDA, the situation is different. General hospital and psychiatric hospital services are both utilized to a high degree. This is not necessarily a miss-allocation as patients with PDA receive professional services in accordance with their disorder. Yet, the question arises whether these services must be delivered in an inpatient setting. It could be worthwhile to develop outpatient-based intervention for PDA to reduce the length of hospital stays [12, 36].

In the outpatient sector, the utilization of mental health services is more pronounce for PDA patients in comparison to PD patients. This in combination with the utilization of inpatient mental health care services could be regarded as indicator for a greater health burden of PDA and hence an increased need for services or a greater awareness of need for support. However, the excess costs for general practitioner (GP) services were comparable for PD and PDA. This and the relatively high costs for GP services (Table 3) could be an indicator for the important part the GP has in the care for PD and PDA patients. This important role of the GP poses a risk and a chance. The risk is that the GP might be not qualified enough to provide or has no access to PD(A)-targeted services. The chance is that the GP as access point for patients into the health care system could be a valuable stakeholder in the care process by providing identification, education and early treatment [12]. Consequently, interventions to empower the GP to identify PD and PDA, to educate the patient and to offer first-line support could be a valuable addition to the services offered by mental health specialists, especially in the German setting, where waiting periods for psychotherapy are quite long [37].

The elevated costs for informal care, can be considered as manifestation of a high degree of need for support. This transfers a part of health care provision into the informal and hence unpaid sector. This relieves the health care budget and promotes the flexibility of service delivery, as family members, friends and neighbours can offer support in a variety of small tasks on short notice. However, there is evidence that the provision of informal care, can lead to worse health and a loss of income on the part of the provider of informal care [38, 39]. This in turn affects the health care budget and societal productivity.

Strengths of our study include the used data sources and the analytical approach. In our study we combined data from a representative population survey [19] with baseline data from a high quality clinical trial with patients diagnosed with panic disorder (with and without agoraphobia) [12]. The combination of these two datasets enabled a valid comparison of panic disorder patients with the general population. In order to reach comparability of the datasets, it is essential to apply elaborated matching or balancing methods. For this reason we applied an innovative reweighting method, Entropy Balancing [28]. In comparison to other matching methods (e.g. propensity score matching), Entropy Balancing is favourable and advantageous [28]. In validation analyses, Entropy Balancing showed a higher degree of balance in covariates in comparison to other common processing adjustments (matching methods), e.g. propensity score or genetic matching [28].

Some limitations should be considered when interpreting our results. As two independent samples where used in this analysis, some data adjustments had to be performed. The assessment of visits to other medical specialist and comorbidities was differently conducted in the datasets. Therefore, data had to be aggregated. Furthermore, the mode of the assessment of comorbidities was different. In the clinical trial general practitioners coded diagnosis, while in the general population sample patients reported their diagnosed comorbidities.

In the main analysis of this study, AF individuals from the general population dataset were compared to individuals with PD and PDA. To determine the population of AF individuals from the general population dataset, individuals with a diagnosed anxiety disorder or with a score of ≥ 3 in the anxiety module of the PHQ-4 [20] were excluded. All other mental comorbidity variables of this AF group were used in the Entropy Balancing in order to create a comparable comparison group to the PD and PDA group in term of mental comorbidities. As anxiety disorders are often diagnosed with other severe mental disorders [40–44], it is possible that the balanced individuals in the AF group had relative mild mental comorbidities and therefore low health care costs in the psychiatric cost categories.

Finally, we want to declare that despite the efforts of mitigating the effects of missing values and differences in sample characteristics by using MICE and entropy balancing, we cannot warrant that the results of our analyses are not influenced by any kind of bias. Entropy balancing led to a high degree of comparability between the groups in measured variables and missing values were imputed by an approach considering uncertainty. However, as both approaches are based exclusively on measured variables, they have no direct influence on unobserved heterogeneity.

## Conclusion

Panic disorder is associated with high excess costs and constitutes a major economic challenge to health care system and society. Resources are mainly utilized in the inpatient sector. The development of measures to identify, educate and treat patients in the outpatient sector could optimize resource allocation. This approach could also lead to a reduction of absenteeism. Further research is needed. Additionally, panic disorder not only affects the patient but also his or her personal/familial network. This could lead to further health and occupational consequences.

## Appendix

See Tables 5, 6.

**Table 5:** 6-month per capita excess cost of PD and PDA for different cost categories in 2012 Euros (complete case analysis)

	AF^a^ group vs. PD group			AF^b^ group vs. PDA group		
	Excess	95% CI	p-value	Excess	95% CI	p-value
Direct costs						
Outpatient sector						
Psychiatrist/neurologist	19	(− 10 to 48)	0.198	15	(3 to 28)	0.019
Psychologist/psychotherapist	89	(19 to 159)	0.013	119	(78 to 159)	< 0.000
General practitioner	129	(104 to 154)	< 0.000	128	(111 to 146)	< 0.000
Other medical specialists	20	(− 21 to 62)	0.331	− 19	(− 51 to 13)	0.243
Inpatient sector						
General hospital	539	(134 to 943)	0.009	261	(4 to 518)	0.046
Psychiatric hospital	0.30	(− 9 to 9)	0.948	239	(32 to 447)	0.024
Rehabilitation hospital	50	(− 41 to 141)	0.422	192	(− 2 to 385)	0.052
Home care sector						
Professional care	− 11	n. a	n. a	− 5	n. a	n. a
Informal care	284	(58 to 510)	0.014	563	(274 to 852)	< 0.000
Total direct cost^a^	782	(150 to 1415)	0.015	840	(337 to 1343)	0.001
Total indirect cost (Absenteeism)	2023	(936 to 3110)	< 0.000	2173	(1482 to 2863)	< 0.000
Total cost	3044	(1368 to 4720)	< 0.000	2933	(1909 to 3957)	< 0.000

AF costs are post balanced estimates

*AF* anxiety free, *PD* panic disorder, *PDA* panic disorder with agoraphobia

^a^AF were balanced on covariate structure of the PD sample

^b^AF were balanced on covariate structure of the PDA sample

**Table 6:** 6-month per capita excess cost of PD and PDA for different cost categories in 2012 Euros (Sensitivity analysis: outlier included)

	AF^a^ group vs. PD group			AF^b^ group vs. PDA group		
	Excess	95% CI	p-value	Excess	95% CI	p-value
Direct costs						
Outpatient sector						
Psychiatrist/neurologist	18	(− 9 to 46)	0.190	13	(0 to 25)	0.044
Psychologist/psychotherapist	94	(25 to 164)	0.008	116	(74 to 157)	< 0.000
General practitioner	128	(103 to 153)	<0.000	137	(117 to 157)	< 0.000
Other medical specialists	20	(− 20 to 59)	0.330	− 14	(− 45 to 18)	0.387
Inpatient sector						
General hospital	619	(134 to 1104)	0.012	306	(− 190 to 802)	0.227
Psychiatric hospital	8	(− 123 to 139)	0.904	298	(− 58 to 654)	0.101
Rehabilitation hospital	131	(− 137 to 400)	0.338	191	(11 to 372)	0.038
Home care sector						
Professional care	− 11	n. a	n. a	− 5	n. a	n. a
Informal care	378	(29 to 727)	0.034	686	(340 to 1031)	< 0.000
Total direct cost^a^	1386	(583 to 2189)	0.001	1733	(819 to 2647)	< 0.000
Total indirect cost (Absenteeism)	2402	(1111 to 3694)	< 0.000	2598	(1763 to 3433)	< 0.000
Total cost	3788	(2076 to 5500)	< 0.000	4331	(2905 to 5757)	< 0.000

AF costs are post balanced estimates

*AF*  anxiety free, *PD* panic disorder, *PDA* panic disorder with agoraphobia

^a^AF were balanced on covariate structure of the PD sample

^b^AF were balanced on covariate structure of the PDA sample

## Abbreviations

AF: anxiety free population
GLM: generalized linear model
PD: panic disorder
PDA: panic disorder with agoraphobia
PHQ: Patient Health Questionnaire
PPP: purchasing power parities
TP: two part model
